# New-Onset Acute Interstitial Nephritis Post-SARS-CoV-2 Infection and COVID-19 Vaccination: A Panoramic Review

**DOI:** 10.1007/s44197-023-00159-4

**Published:** 2023-10-23

**Authors:** Yu Wang, Ling Yang, Gaosi Xu

**Affiliations:** 1 Department of Nephrology, Donghu District, the Second Affiliated Hospital of Nanchang University, No. 1, Minde Road, Nanchang, 330006 Jiangxi China; 2 Department of Health Management Medicine, The Second Affiliated Hospital of Nanchang University, Jiangxi, China

**Keywords:** Acute interstitial nephritis, COVID-19, SARS-CoV-2, Vaccination

## Abstract

The 2019 coronavirus disease (COVID-19) pandemic caused by severe acute respiratory syndrome coronavirus type 2 (SARS-CoV-2) has posed a considerable challenge to global healthcare. Acute interstitial nephritis (AIN) post SARS-CoV-2 infection and vaccination has been reported, but its clinical features and pathogenesis remained unclear. We reviewed so far the largest 22 cases of AIN post SARS-CoV-2 infection and 36 cases of AIN following COVID-19 vaccination. The onset of AIN was mainly related to messenger RNA vaccines (52.8%). Apart from fever, proteinuria (45.5%) was the main manifestation of AIN post SARS-CoV-2 infection, left acute kidney injury (AKI, 63.9%) in patients post COVID-19 vaccination. The potential mechanism of vaccination induced AIN was conjugating vaccines with proteins to form a hapten, which activated dendritic cells and promoted a cascade immunological reaction leading to AIN.

## Introduction

Coronavirus disease 2019 (COVID-19), caused by severe acute respiratory syndrome coronavirus 2 (SARS-CoV-2), was one of the deadliest viral epidemics in human history, posing a serious threat to human health and economic development [1]. Due to the wide distribution of angiotensin-converting enzyme 2 (ACE2) receptors, the gastrointestinal tract, kidney, and liver were often involved, with more severe clinical symptoms and higher mortality [2, 3]. COVID-19 vaccination was one of the key strategies for controlling the disease. After vaccination, the innate and adaptive immune systems would be initiated by the vaccine itself or by vaccine adjuvants, producing protective antibodies [4, 5], and based on previous evidence, most adverse events following vaccination were nonserious, such as fatigue, headache, and myalgia [6]. However, with widespread vaccination, acute interstitial nephritis (AIN) has been reported.

AIN was characterized by the presence of inflammatory infiltrates and edema within the interstitium, usually associated with an acute deterioration in renal function. AIN was one of the common causes of acute kidney injury (AKI) from biopsy samples [7]. Although the hapten formation was thought to be the key process that triggered the immune response, the exact mechanisms of AIN post SARS-CoV-2 infection and COVID-19 vaccination were unclear [8].

In the present analysis, we summarized the clinical evidence of AIN following the SARS-CoV-2 infection and COVID-19 vaccination published by September 9, 2023, with the largest sample size, and analyzed the clinical characteristics of the included cases.

## Methods

In this review, we searched relevant literature on AIN post SARS-CoV-2 infection or vaccination through electronic databases, including PubMed, EMBASE, and Web of Science, using keywords (“Interstitial Nephritis” OR “Interstitial Nephritides” OR “Tubulointerstitial Nephritides” OR “Tubulointerstitial Nephritis”) AND (“COVID-19” OR “Novel Corona Virus” OR “Coronavirus” OR “2019-nCoV” OR “SARS-CoV-2”) or (“Interstitial Nephritis” OR “Interstitial Nephritides” OR “Nephritides, Tubulointerstitial” OR “Tubulointerstitial Nephritides” OR “Tubulointerstitial Nephritis”) AND (“COVID-19” OR “Novel Corona Virus” OR “Coronavirus” OR “2019-nCoV” OR “SARS-CoV-2”) AND (“Vaccines” OR “Vaccination”).

We reported medians and ranges for continuous data and numbers and percentages for categorical data. We used descriptive statistics in this report and performed statistical analysis. The Mann–Whitney Test was used for continuous data, and the Chi-Square Test was used for categorical data to determine whether the two groups were statistically different. Since our sample size was less than 40, the Fisher's Exact Test was used for both simple four-table and R × C table data. All statistical analyses were performed using SPSS 25.0 software, and *P* value < 0.05 was considered to be statistically significant.

## Results

### Baseline Demographic and Clinical Characteristics of Patients with AIN Post-SARS-CoV-2 Infection

There were 22 patients appeared with AIN after SARS-CoV-2 infection [9–24] (Table 1), including 10 (45.5%) tubulointerstitial nephritis and uveitis (TINU) [16, 20, 23, 24]. The median age was 15 (10–78) years old, and 68.2% (15 of 22) of the patients were male. The majority of patients were European (68.2%), followed by Asian (18.2%). The median time from SARS-CoV-2 infection to AIN was 14.5 (1–37) days.

**Table 1:** Summary of published cases of acute interstitial nephritis following SARS-CoV-2 infection

Case	Authors/ Country (Race)	Age/Sex	Medical history	Baseline (SCr)	COVID-19 symptoms (diagnosis)	Time to AIN symptom onset	Symptoms	Urinalysis	Blood test	Renal biopsy	Treatment	Outcomes
1 [9]	Tuma J et al./Swiss	78/M	NA	SCr:50-100µmol/L	RT-PCR ( +)	22 days	NRP, fever, abdominal pain, loss of appetite	UTP:04 g/d, eGFR:20ml/min,	SCr:304µmol/L, CRP:180 mg/L (peak)	NA	Vancomycin	R SCr less than 100µmol/L
2 [10]	Azukaitis K et al./ Czechia	12/M	None	NA	RT-PCR ( +)	1 week	Fatigue, anorexia, polydipsia, general malaise, increased thirst, anemia, glucosuria, low-molecular-weight proteinuria, mild leukocyturia, microhematuria	Glucosuria:3 + , Proteinuria:1.5 g/L, Leukocyturia:1 + , Hematuria:3 +	SCr:235µmol/L (W7), 272µmol/L (peak), CRP:49.6 mg/L	AIN, IHC staining for SARS- CoV-2 ( +)	Steroid	R eGFR was 106 ml/min and isolated microhematuria at the follow up
3 [11]	Buyansky D et al./ Canada	62/F	Poorly differentiated squamous cell carcinoma with lymph node metastases and peritoneal carcinomatosis (treatment with durvalumab)	SCr:50µmol/L	RT-PCR ( +)	10 days	AKI, hyperkalemia, leukocyturia, microscopic hematuria, proteinuria, dry cough, dyspnea, fever	UPCR:0.441 g/mmol WBC:11–100/HPF, RBC:11–100/HPF	SCr:500µmol/L CRP:271.5 mg/L	ATIN	Hemodialysis, Steroid	R Kidney function returned to her baseline levels
4 [12]	León-Román J et al./Spain	64/M	NA	SCr:80µmol/L	Pneumonia	1 day	AKI, shortness of breath, fever	UPCR:1.7 g/g, ACR:0.13 g/g	SCr:212µmol/L (D7)	AIN	ET, CVVHDF, Steroid Antibiotic	CR SCr was 71µmol/L at 3 months of follow-up
5 [13]	Alotaibi M et al./USA	52/M	MS, CKD	SCr:141µmol/L	Pneumonia RT-PCR ( +)	NA	Fever, cough, fatigue, oliguric kidney failure, proteinuria, hematuria	UPCR > 2000/39.1 g/g, RBC:4–10/HPF,	SCr:849µmol/L, SCr:1211µmol/L (D4), ALB:25 g/L, CRP:122 mg/L	GIN, COVAN, ATN	Hemodialysis, High-dose steroid, Remdesivir, Losartan/Hctz	R Kidney function back to previous baseline, but proteinuria did not remission
6 [14]	Szajek K et al./ Caucasian	62/M	Hyperlipidemia	SCr:87µmol/L	Pneumonia	13 days	AKI, ARDS, multi-organ failure, fever, cough, myalgia, chills, atrial fibrillation, paralytic ileus, hemolytic anemia, maculopapular rash, leukocyturia, microscopic hematuria	UPCR:72.6 mg/mmol, ACR:5.3 mg/mmol	SCr:498µmol/L, 519µmol/L (peak)	GIN, AIN	CVVHDF, Steroid, Hydroxychloroquine, Antibiotic,	R Improvement of kidney function and translation to chronic kidney disease (eGFR was 43 ml/min at two months of discharge)
7 [15]	Serafinelli J et al./ Italy	12/F	None	NA	Mild respiratory manifestations	4–6 weeks	Weakness, weight loss, hypouricemia, anorexia, ketonuria proteinuria, glycosuria	UPCR:0.5 mg/mg, eGFR:59ml/min,	SCr:159µmol/L	AIN	Steroid	CR Complete recovery in 2 weeks
8 [16]	EserOzturk H et al./ Turkey	17/M	NA	NA	Pneumonia	NA	SRP, weight loss, fatigue, photo- phobia, redness, and decreased vision in the right eye	UTP:0.5 g/d	SCr:304µmol/L	TINU	Steroid, ADA, MTX	R
9 [16]	EserOzturk H et al./ Turkey	13/M	NA	NA	NA	15 days	SRP, malaise, weight loss, fever, headache, cough	UTP:1 g/d	SCr:273µmol/L, CRP:122 mg/dL	TINU	Steroid, MTX	R
10 [16]	EserOzturk H et al./ Turkey	14/F	NA	NA	NA	2 weeks	Weakness, chest pain, flank pain, redness and blurred vision in the left eye	NA	SCr:159µmol/L	TINU	Steroid	R
11 [16]	EserOzturk H et al./ Turkey	16/M	NA	NA	RT-PCR ( +)	15 days	SRP, cough, weakness, loss of appetite, weight loss, redness and blurred vision in the left eye	UTP:0.4 g/d	SCr:181µmol/L	TINU	Steroid	R
12 [17]	Westhoff TH et al./ Germany	69/M	Pancreas-kidney transplantation	NA	Pneumonia RT-PCR ( +)	NA	Seizures, fever, cough, diarrhea, meningoencephalitis	NA	NA	AIN RT-PCR ( +)	Steroid	R SCr was 106µmol/L
13 [18]	SobieszczańskaDroździel A et al./ Poland	10/M	NA	NA	RT-PCR ( +)	a few weeks	Weakness, fatigue, weight loss, polyuria	eGFR:58.3 ml/min	SCr:124µmol/L, CRP:4.42 mg/Dl, ESR:131 mm/h, Hb:103 g/L, Ferritin:307.71ug/L	NA	Steroid	CR Normalization of kidney function
14 [19]	Nikolova M et al./ Bulgaria	44/M	NA	NA	COVID rapid antigen ( +)	7 days	ARF, arthralgia, fever, diarrhea,	Proteinuria:0.11 g/L	SCr:399µmol/L, CRP:49 mg/dL, ESR:68 mm/h, Leukocytosis:11.7 G/l, Eosinophil:0.53 G/l	AIN	Steroid	CR
15 [20]	Bilak VM et al./Ukraine	11/M	NA	NA	RT-PCR ( +)	NA	Abdominal pain, subfebrile temperature, weakness, loss of appetite	Albuminuria:150 mg/L, Glucosuria:148 mg/dl	SCr:56.8mkmol/L CRP:1.59 mg/dL, ESR:38 mm/h,	TINU	Steroid	R SCr was 52.6mkmol/L
16 [21]	Nowak PJ et al./ Poland	34/M	HTN, anabolic steroids, and high-protein diet	SCr:113µmol/L	rRT-PCR ( +)	NA	NRP, diarrhea, headache, inter- mittent pain in the right hypochondriac and both lumbar re-gions, fever, cough, dyspnea on exertion, fatigue, anosmia, ageusia	UTP:22 g/d, Proteinuria:10.2 g/L, ACR:1982 mg/g	SCr:520µmol/L CRP:300 mg/L	FSGS AIN	Steroid, Cyclosporine A	R UTP was 0.4 g/d
17 [22]	Lebedeva MV et al./ Russia	64/M	HTN, overweight, sarcoidosis of the lungs and intrathoracic lymph nodes, mechanical aortic valve repair, coronary artery bypass surgery	Normal	Pneumonia	37 days	SRP, fever, weakness, breathing difficulties, edema	UTP:1 g/d, RBC:15–20/HPF	SCr:231-239µmol/	GIN	Steroid	R SCr was 112µmol/L after 4 months
18 [23]	Sakhinia F et al./ UK	12/F	NA	NA	SARS-CoV-2 Antibody ( +)	NA	Abdominal pain, constipation, anorexia, weight loss, vomiting, fatigue, pyrexia, glycosuria, proteinuria	eGFR:19.2ml/min, ACR:25.8 mg/mmol, UPCR:167 mg/mmol,	ESR:141 mm/H, HB:84 g/L	TINU	MMF, Prednisolone Acetate	R eGFR, ACR and UPCR were 120ml/min,21.6 mg/mmol, and 11 mg/mmol at followed up
19 [23]	Sakhinia F et al./ UK	11/M	NA	NA	SARS-CoV-2 Antibody ( +)	NA	Abdominal pain, constipation, anorexia, weight loss, vomiting, pyrexia, polyuria, polydipsia, glycosuria, proteinuria	eGFR:69.3ml/min, ACR:7.1 mg/mmol, UPCR:117 mg/mmol,	ESR:124 mm/H, HB:104 g/L	TINU	MMF, Prednisolone Acetate	R eGFR, ACR and UPCR were 84.1ml/min,18.5 mg/mmol, and 68 mg/mmol at followed up
20 [23]	Sakhinia F et al./ UK	14/F	NA	NA	SARS-CoV-2 Antibody ( +)	NA	Fatigue, glycosuria, proteinuria	eGFR:42.8ml/min, ACR:16.3 mg/mmol,	ESR:16 mm/H, HB:105 g/L	TINU	MMF, Prednisolone Acetate	R eGFR, ACR and UPCR were 150.5ml/min,1.8 mg/mmol, and 41 mg/mmol at followed up
21 [23]	Sakhinia F et al./ UK	14/F	NA	NA	A history of COVID-19 contact	NA	Abdominal pain, constipation, joint pain, glycosuria, proteinuria	eGFR:57.9ml/min, ACR:9.4 mg/mmol, UPCR:68 mg/mmol,	ESR:30 mm/H, HB:121 g/L	TINU	MMF, Prednisolone Acetate	R eGFR, ACR and UPCR were 84.7ml/min,5.7 mg/mmol, and 13 mg/mmol at followed up
22 [24]	Maggio MC et al./ Italy	14/F	NA	NA	RT-PCR ( +)	28 days	Nocturia, polyuria, polydipsia, fever, bilateral red eyes, photophobia, eye pain, glycosuria, albuminuria, proteinuria	eGFR:60ml/min, β2-microglobulin:6.4 mg/L, α1-microglobulin:31.6 mg/L	NA	NA	Steroid, Adalimumab	R SCr and eGFR normalized at followed up

COVID-19, coronavirus disease 2019; F, female; M, male; SCr, serum creatinine; AIN, acute interstitial nephritis; GIN, granulomatous interstitial nephritis; AKI, acute kidney injure; ATN, acute tubular necrosis; ATI, ARF, acute tubular injury, acute renal failure; ET, endotracheal intubation; CVVHDF, continuous veno-venous hemodiafiltration; MS, multiple sclerosis; HTN, hypertension; CKD, chronic kidney disease; Hctz, hydrochlorothiazide; COVAN, collapsing glomerulopathy; UACR, urinary albumin-creatinine ratio UPCR, urine protein-to-creatinine ratio; ACR, micro-albumin-creatinine ratio; ALB, serum albumin; CRP, c-reactive protein; CR, complete remission; PR, partial remission; NA, non-applicable; NR, no response; R, response; RBC, red blood cell; WBC, white blood cell; HPF, high power field; USA, the United States of America; ARDS, acute respiratory distress syndrome, ADA: Adalimumab; MTX: Methotrexate; UTP, 24-h urine protein; TINU, tubulointerstitial nephritis and uveitis; SRP, sub-nephrotic range proteinuria; NRP, nephrotic range proteinuria; IHC, immunohistochemistry; FSGS, focal segmental glomerulosclerosis; MMF mycophenolate mofetil; Hb, hemoglobin

Of the 22 patients, 4 received renal replacement therapy and immunotherapy, 17 received immunosuppressive therapy, and only 1 received conservative therapy. Of the available follow-up data, 21 patients responded well to treatment. Case 6 [14] progressed to chronic kidney disease despite some recovery of renal function (Table 2).

**Table 2:** Clinical characteristics of patients with acute interstitial nephritis post SARS-CoV-2 infection

Characteristics	COVID-19 vaccination (n = 36)	SARS-CoV-2 infection (n = 22)	*P*
Age (yr)	55 (12–78)	15 (10–78)	0.001^a^
Male sex, n (%)	17 (47.2)	15 (68.2)	0.174
Geographic region,			0.293
Asia	8 (22.2)	4 (18.2)	
Europe	17 (47.2)	15 (68.2)	
United Stated	9 (25)	2 (9.1)	
Africa	1 (2.8)	0	
Oceania	1 (2.8)	0	
North America	0	1 (4.5)	
Time of symptom onset, days			0.401
Cases	9.5 (1–82)	14.5 (1–37)	
Time of symptom onset, n (%)			0.329
< 7days	11 (30.6)	1 (4.5)	
7–10 days	7 (19.4)	3 (13.6)	
> 10days	18 (50)	8 (36.4)	
Symptoms, n (%)			< 0.001^a^
Acute kidney injury	23 (63.9)	3 (13.6)	
Hematuria	12 (33.3)	4 (18.2)	
Proteinuria	16 (44.4)	10 (45.5)	
Fever	4 (11.1)	13 (59.1)	
Pain	6 (16.7)	8 (36.4)	
Leukocyturia	9 (25)	3 (13.6)	
Blood test			0.186
SCr	355 (86–1679)	239 (52–849)	
Treatment, n (%)			0.529
Steroid	24 (66.7)	17 (77.3)	
Hemodialysis + Steroid	10 (27.8)	4 (18.2)	
Outcomes			0.514
Response	32 (88.9)	22 (100)	
Not response	2 (5.6)	0	

^a^Statistically different

### Baseline Demographic and Clinical Characteristics of Patients with AIN Post-COVID-19 Vaccination

A total of 36 patients were diagnosed with AIN following COVID-19 vaccination [25–46] (Table 3), 35 of which were confirmed by renal biopsy, and all patients were first diagnosed. The majority of patients were European (47.2%), followed by Americans (25%) and Asians (22.2%). In addition, 52.8% of the patients received the BNT162b2 (Pfizer) vaccine, 22.2% received the mRNA-1273 (Moderna) vaccine, 19.4% received the AstraZeneca vaccine, and another 5.6% received the inactivated (Sinovac) vaccine. Common clinical presentations were AKI, proteinuria, hematuria, and leukocyturia. The median serum creatinine value was 355 (86—1679) μmol/L. Follow-up data were obtained for 35 patients, with 32 responding well to treatment (Table 4).

**Table 3:** Summary of published cases of acute interstitial nephritis following COVID-19 vaccination

Case	Authors/ Country (Race)	Age/Sex	Medical History	Baseline (SCr)	Vaccine	Timing of Symptom Onset	Vaccine Dose	Symptoms	Urinalysis (Hematuria/ Proteinuria)	Blood Test	Renal Biopsy	Treatments Received	Outcomes
1 [25]	Dheir H et al./ Turkey	44/F	None	NA	mRNA (Pfizer)	2 days	First	AKI, HA, NV, NRP, anemia fever, minimal leukocyturia, micro-hematuria, lower extremities pain, weakness,	UTP:10.1g//d CRP:96.2mg/dl	DCT ( +) SCr:186μmol/L (D1) 575μmol/L (D2) HB:66g/L	AIN	Steroid + Hemodialysis + BT	CR Full clinical recover but abnormal urinalysis
2 [26]	Fenoglio R et al./Italy	78/F	NA	Normal	mRNA (Pfizer)	52 days	First	RF, UA	NA	NA	AIN	Dialysis + Steroid	R Dialysis discontinuation after 2 months
3 [27]	de la Flor et al./Spain	78/M	CKD (3a-b), HTN, T2DM, dyslipidemia, hyperuricemia	SCr:150μmol/L eGFR:39mL/mi ACR:1.4mg/g	mRNA (Pfizer)	3 weeks	First	SRP, anemia, hematuria, mild dehydration, eosinophilia, leukocyturia	UTP:3.4g//d ACR:3.397mg/g	SCr:476μmol/L Urea:156 mg/dL	AIN	Hemodialysis + Steroid	NR Remained dialysis-dependent
4 [28]	Wu HHL et al./UK	69/F	RA, SS, HTN, hypothyroidism, anxiety	SCr:85μmol/L	Adenovirus vector (AstraZeneca)	5 days	First	AKI, diuresis, eosinophilia	Normal	SCr:245mol/L (D1), 90μmol/L, 250μmol/L (M1), 130μmol/L (M2)	AIN	Steroid + Discontinue ramipril lansoprazole methotrexate and paroxetine	R SCr:130μmol/L peripheral eosinophilia reduction
5 [29]	Klomjit N et al./USA	44/M	NA	SCr:93μmol/L	mRNA (Moderna)	2 weeks	First	AKI, NRP	RBC:21–30/HPF UTP:14g/d	SCr:221μmol/L ALB:37g/L	IgAN AIN	High-dose steroid	NR SCr, RBC and UTP were318μmol/L,3–10/HPF and5.6g/d within 3months
6 [30]	Tan, FS et al./ UK	70/F	T2DM, HTN, DR	SCr:80μmol/L	Adenovirus vector (AstraZeneca)	8 days	First	AKI, HA, hyperkalemia, metabolic acidosis, hematuria, weakness, proteinuria	UPCR:135mg/mmol	SCr:416μmol/L, 618μmol/L (peak) HB:97g/L K^+^:6.1 mmol/L Blood sugar: 1.6mmol/L	AIN	Steroid	R SCr were 234μmol/L and183μmol/L at 2 weeks and 4 weeks post discharge
7 [31]	Rieckman S et al./Germany	63/M	None	Normal	mRNA (Pfizer)	3 weeks	First	AKI, hematuria, proteinuria, anuria, metabolic acidosis, hyperkalemia, leukocyturia	ACR:393mg/g UPCR:787mg/g	SCr:1679μmol/L	AIN ATN	Hemodialysis + Steroid	R Hemodialysis was discontinued after 2 weeks
8 [32]	Missoum S et al./ Algeria	58/M	HTN	NA	Inactivate (Sinovac)	9 days	First	AKI, SRP, fever, arthralgia, vascular purpura, nausea, leukocyturia, hematuria	UTP:0.24g/d	SCr:791μmol/L, CRP:50 mg/L, ESR:86 mm/h, WBC:14.5*10^9^/L	AIN	Hemodialysis + Steroid	R SCr was 248μmol/L at 3 months
9 [33]	Jongvilaikasem P and Rianthavorn P/ Thailand	14/M	None	NA	mRNA (Pfizer)	5 days	First	AKI, HTN, NS, edema, anuria, proteinuria	UPCR:9g/g	SCr:177μmol/L, 795.6μmol/L (peak) ALB:20 g/L	AIN MCD	Steroid + Hemodialysis for 3 weeks	R SCr and UPCR were 47 μmol/L and 0.9 g/g after 5 weeks
10 [34]	Lim JH et al./ Korea	44/M	T2DM, chronic hepatitis B, hyperlipidemia	SCr:80.4μmol/L	mRNA (Moderna)	1 day	First	AKI, anorexia, proteinuria, gastrointestinal discomfort	UPCR:1.01g/g	SCr:365 μmol/L (W1), 436.7μmol/L (W3)	AIN	High-dose steroid	R SCr and UPCR were 167μmol/L and 0.3 g/g after 6 weeks
11 [35]	Baker R et al /USA	55/M	Metastatic lung adenocarcinoma	SCr:97–115 μmol/L	mRNA (Pfizer)	7 days	First	AKI, fatigue, myalgia, leukocyturia, hematuria	UPCR:0.09 g/g Leukocytes:5–10/HPF RBC:2/HPF	SCr:676.3 μmol/L (W1) 714.3μmol/L (peak)	AIN	Pembrolizumab was discontinued + Steroid	R SCr was 150μmol/L after 6 months
12 [35]	Baker R et al /USA	68/F	Metastatic melanoma	SCr:115–133 μmol/L	mRNA (Pfizer)	10 days	First	Fatigue	UPCR:0.1 g/g	SCr:301μmol/L	AIN	Lpilimumab was discontinued + Steroid	NA He was hospitalized in the 5th month of follow-up for infectious shock due to pneumonia and died of multi-organ failure
13 [26]	Fenoglio R et al./Italy	57/F	NA	NA	mRNA (Pfizer)	82 days	Second	RF, UA	NA	NA	AIN	Steroid	NA
14 [26]	Fenoglio R et al./Italy	65/F	NA	NA	Adenovirus vector (AstraZeneca)	24 days	Second	RF, UA	NA	NA	AIN	Dialysis + Steroid	R Dialysis discontinuation after 1 months
15 [28]	Wu HHL et al./UK	60/F	HTN	SCr:59μmol/L	Adenovirus vector (AstraZeneca)	2 weeks	Second	AKI, generally unwell, tubular proteinuria	UACR:20mmol/μmol UPCR:160 mmol/μmol	SCr:754μmol/L	AIN	Steroid	CR SCr was 216μmol/L in last follow-up review
16 [31]	Rieckmann S et al./Germany	18/M	None	NA	mRNA (Pfizer)	6 weeks	Second	Fatigue, cough, proteinuria, hematuria, glycosuria	ACR:393mg/g, UPCR:787mg/g	SCr:150μmol/L	AIN ATN	Steroid	CR
17 [34]	Lim JH et al./ Korea	77/F	T2DM, chronic hepatitis B, hepatocellular carcinoma	SCr:86.6μmol/L	mRNA (Pfizer)	1 day	Second	AKI, NV, anorexia	UPCR:4.63g/g	SCr:950 μmol/L (W1), 985.7μmol/L (W2), CK:381U/L, LDH:427U/L, MB:1180 ng/mL	AIN MB tubular casts	Low-dose steroid + Hemodialysis	R SCr was 187.4μmol/L after 4 months
18 [36]	Czerlau C et al./ Switzerland	55/M	HTN, prostate cancer treated with prostatectomy	SCr:76.5μmol/L	mRNA (Pfizer)	4 days	Second	AKI, NRP, renal pain	UTP:11 g/d, Proteinuria:8.3g/L, Albuminuria:1251 g/molcr, Glomerular Erythrocytes:4%	SCr:355μmol/L	AIN	Steroid	R SCr was 88μmol/L
19 [36]	Czerlau C et al./ Switzerland	54/M	Myocardial infarction	NA	mRNA (Moderna)	3 days	Second	NS, glomerular hematuria	Proteinuria:9.7g/L Albuminuria:994 g/molcr Glomerular erythrocytes:25%	SCr:268μmol/L	AIN, FSGS	Steroid	R SCr was 235μmol/L
20 [36]	Czerlau C et al./ Switzerland	58/M	FSGS refractory to treatment with multiple relapses	SCr:167μmol/L	mRNA (Moderna)	A few days	Second	AKI, NS, edema aggravated	Proteinuria:3.2g/L Albuminuria:886 g/molcr	SCr:355μmol/L	AIN, FSGS	Steroid	R SCr was 210μmol/L
21 [36]	Czerlau C et al./ Switzerland	38/F	Ulcerative colitis	SCr:76μmol/L	mRNA (Moderna)	1 month	Second	SRP	UTP:0.5 g/d Proteinuria:0.61g/L Albuminuria:26 g/molcr Glomerular erythrocytes:56%	SCr:86μmol/L	AIN	Steroid	R SCr was 72μmol/L
22 [36]	Czerlau C et al./ Switzerland	35/F	RA (treatment with certolizumab)	SCr:49μmol/L	mRNA (Pfizer)	Exact time not specified	Second	SRP, unwell, tired	Proteinuria:2g//L Glomerular Erythrocytes:12%	SCr:100μmol/L	AIN	Steroid	R SCr was 90μmol/L
23 [37]	Unver S et al./ Turkey	67/F	T2DM, recent new onset MCD following first dose of CoronaVac	SCr:71μmol/L	Inactivate (CoronaVac)	1 week	Second	AKI, NRP, HA, HTN, hematuria, swell, gain weight, leukocyturia,	UTP:18.6g//d	SCr:371μmol/L, 459.7μmol/L (peak) TP:40g/L, ALB:15g/L, Urea:102 mg/dL,	AIN	Steroid + Cyclosporine	R SCr and UTP were 99μmol/L and 3 g/d at the last follow-up
24 [38]	Mira et al./ Caucasian	45/F	Multinodular goiter treatment by total thyroidectomy	SCr:75μmol/L	mRNA (Pfizer)	1 day	Second	AKI, NV, SRP, anorexia, hematuria, leukocyturia anemia, hyperkalemia, metabolic acidosis, oliguria, macular rash on lower limbs and palms	UTP:0.5g/d	SCr:1626.6μmol/L 389μmol/L (D15) ESR:58 mm/h LDH:410U/L, CRP:1.8 mg/dl, ALB:31 g/L	AIN ATN	Hemodialysis + Steroid	CR SCr was 90.2μmol/L During the follow-up
25 [39]	Choi JH et al./South Korea	17/M	None	NA	mRNA (Pfizer)	1 day	Second	AKI, HTN, epigastric pain, poor oral intake, myalgia, fever, nausea	Normal	SCr:265μmol/L, ESR:43 mm/h, CRP:3.23 mg/dL	AIN	Supportive care	R Discharged after 1 week
26 [39]	Choi JH et al./South Korea	12/M	None	NA	mRNA (Pfizer)	1 day	Second	AKI, NV, SRP, anorexia, loss of weight, dehydration, metabolic acidosis, pyuria	UTP:0.861 g/d Leukocytes:10–19/ HPF, casts > 21/LPF, UPCR:1.95 g/g	SCr:199μmol/L CRP:6.05 mg/dL	AIN	Steroid	R
27 [40]	Caza TN et al./USA	73/M	NA	NA	mRNA (Pfizer)	2 weeks	Second	AKI, hematuria	UTP:0.5g/d	SCr:300μmol/L	AIN IgAN	Steroid	R SCr and UTP were 8.4 μmol/L and 2.4 g/d at follow up
28 [41]	Liew, SK et al./UK	53/M	HTN	NA	Adenovirus vector (AstraZeneca)	3 days	Second	AKI, SRP, flank pain	Proteinuria:0.6g/L	SCr:1034μmol/L CRP:141mg/L	AIN	Dialysis + Steroid	R Dialysis-independent and SCr was 226μmol/L when last follow up
29 [42]	Schaubschlager T et al./USA	60/F	HTN, diffuse large B-cell lymphoma-In remission since 2019, hypothyroidism	SCr:79μmol/L	mRNA (Moderna)	4 weeks	Second	AKI, hematuria, cough, shortness of breath	UPCR:2g/g	SCr:401μmol/L, 435μmol/L (peak), C3:53 mg/dL	AIN, GN	Steroid + MMF	R SCr and UPCR were 132.6μmol/L and 0.2 g/g at last follow up
30 [42]	Schaubschlager T et al./USA	39/F	SLE, hashimoto thyroiditis	SCr:150μmol/L	mRNA (Moderna)	4 weeks	Second	AKI, SRP, leukocyturia	UPCR:0.5g/g UPCR:2.5g/g	SCr:389μmol/L 495μmol/L (peak)	AIN, GN class II LN	Steroid + MMF	R SCr and UPCR were 247.5μmol/L and 0.3 g/g at last follow up
31 [43]	Baskaran K et al./Australia	55/M	NA	NA	Adenovirus vector (AstraZeneca)	1 week	Second	AKI, oliguria, ascites, peripheral edema	UPCR:1631 mg/mmol	SCr:633μmol/L ALB:18 g/L	AIN MCD ATN	Steroid	R
32 [44]	Nakamura H et al./ Japan	74/M	Hyperlipidemia	SCr:66.3μmol/L	mRNA (Pfizer)	48 days	Second	RF, hematuria	RBC:100/HPF, WBC:5–9/HPF	SCr:115.8μmol/L, C3:50.1 mg/dL	TIN DDD	Steroid	R Gradually improved
33 [45]	Williams SBM et al./UK	51/F	None	NA	Adenovirus vector (AstraZeneca)	7 days	Second	AKI, HTN, weight loss, fatigue, proteinuria, hematuria, nausea, metallic taste	UPCR:50 mg/mmol	SCr:484μmol/L 626μmol/L (peak) CRP:77mg/Dl C3:1.89 g/L	AIN	Steroid	R SCr was 78μmol/L at last follow up
34 [31]	Rieckmann S et al./Germany	25/F	None	NA	mRNA (Pfizer)	3 weeks	Third	AKI hematuria, proteinuria, general malaise, fatigue glycosuria	ACR:58mg/g UPCR:235mg/g	SCr:1034μmol/L	AIN, ATN	Steroid	R
35 [35]	Baker R et al /USA	65/F	Invasive bladder neoplasm	SCr:71–97 μmol/L	mRNA (Pfizer)	1 month	Third	None	UPCR:0.2 g/g uRBP/Cr: 3797mcg/g	SCr:192.7μmol/L CRP:40mg/dL	NA	Pembrolizumab was discontinued	R CRP < 3 mg/dL and SCr was 106μmol/L after 6 weeks
36 [46]	Ghanekar K et al./USA	73/F	HTN, seasonal Allergies, hyperlipidemia, hypothyroidism	SCr:70.7μmol/L	mRNA (Moderna)	3 weeks	Third	AKI, leukocyturia, eosinophilia,	WBC:21–30/HPF	SCr:114.9μmol/L SCr:482.7μmol/L (peak)	ATIN	Steroid	R SCr was 150.3μmol/L at last follow up

*COVID-19* coronavirus disease 2019, *F* female, *M* male, *SCr* serum creatinine, *AIN* acute interstitial nephritis, *eGFR* estimated glomerular filtration rate, *USA* the United States of America, *AKI* acute kidney injure, *CRP* c-reactive protein, *TP* total protein, *ALB* serum albumin, *UACR* urinary albumin-creatinine ratio, *UPCR* urine protein-to-creatinine ratio, *ACR* micro-albumin-creatinine ratio, *T2DM* type II diabetes mellitus, *CR* complete remission, *PR* partial remission, *NA* non-applicable, *NR* no response, *R* response, *RF* renal failure, *UA* urinary abnormalities, *NS* nephrotic syndrome, *SS* sjogren’s syndrome, *LN* lupus nephritis, *SLE* systemic lupus erythematosus, *IgAN* IgA nephropathy, *ATN* acute tubular necrosis, *MCD* minimal change disease, *FSGS* focal segmental glomerulosclerosis, *RBC* red blood cell, *C3* complement C3, *HTN* hypertension, *CKD* chronic kidney disease, *ALB* serum albumin, *SRP* sub-nephrotic range proteinuria, *NRP* nephrotic range proteinuria, *UTP* 24-h urine protein, *HD* hemodialysis, *HA* headache, *BT* blood transfusion, *NV* nausea-vomiting, *DCT* direct coombs testing, *RA* rheumatoid arthritis, *DR* diabetic retinopathy, *ESR* erythrocyte sedimentation rate, *WBC* white blood cell, *HPF* high power field, *LPF* low power field, *CK* creatine phosphokinase, *LDH* lactate dehydrogenase, *Mb* myoglobin, *uRBP/Cr* urine retinol binding protein to creatinine ratio, *MMF* mycophenolate mofetil, *GN* glomerulonephritis

**Table 4:** Clinical characteristics of patients with acute interstitial nephritis post COVID-19 vaccination

Characteristics	First dose (n = 12)	Second dose (n = 21)	Third dose (n = 3)	Total (n = 36)	*P*
Age (yr)	60.5 (14–78)	55 (12–77)	65 (25–73)	55 (12–78)	0.269
Male sex, n (%)	7 (58.3)	10 (47.6)	0	17 (47.2)	0.721
Geographic region,					0.770
Asia	3 (25)	5 (23.8)	–	8 (22.2)	–
Europe	5 (41.7)	11 (52.4)	1 (33.3)	17 (47.2)	–
United Stated	3 (25)	4 (19)	2 (66.7)	9 (25)	–
Africa	1 (8.3)	0	–	1 (2.8)	–
Oceania	0	1 (4.8)	0	1 (2.8)	
Medical history					–
hypertension	4 (33.3)	4 (19)	1 (33.3)	9 (25)	–
Type 2 diabetes mellitus	3 (25)	2 (9.5)	–	5 (13.9)	–
Vaccine type, n (%)					0.897
BNT162b2 (Pfizer)	7 (58.3)	10 (47.6)	2 (66.7)	19 (52.8)	
mRNA-1273 (Moderna)	2 (16.7)	5 (23.8)	1 (33.3)	8 (22.2)	
Adenovirus vector (AstraZeneca)	2 (16.7)	5 (23.8)	–	7 (19.4)	
Inactivated (Sinovac)	1 (8.3)	1 (4.8)	–	2 (5.6)	
Time of symptom onset, days					0.919
Cases	8.5 (1–52)	7 (1–82)	21 (21–30)	9.5 (1–82)	
Time of symptom onset, n (%)					0.421
< 7days	4 (33.3)	7 (33.3)	–	11 (30.6)	
7–10 days	4 (33.3)	3 (14.3)	–	7 (19.4)	
> 10days	4 (33.3)	11 (52.4)	3 (100)	18 (50)	
Symptoms, n (%)					0.900
Acute kidney injury	9 (75)	12 (57.1)	2 (66.7)	23 (63.9)	
Hematuria	6 (50)	5 (23.8)	1 (33.3)	12 (33.3)	
Proteinuria	8 (66.7)	7 (33.3)	1 (33.3)	16 (44.4)	
Fever	2 (16.7)	2 (9.5)	0	4 (11.1)	
Pain	2 (16.7)	4 (19)	0	6 (16.7)	
Leukocyturia	5 (41.7)	3 (14.3)	1 (33.3)	9 (25)	
Blood test					0.747
SCr	365 (177–1679)	355 (86–1626.6)	192.7 (114.9–1034)	355 (86–1679)	
Treatment, n (%)					0.119
Steroid	6 (50)	16 (76.2)	2 (66.7)	24 (66.7)	
Hemodialysis + Steroid	6 (50)	4 (19)	–	10 (27.8)	
Outcomes					0.118
Response	9 (75)	20 (95.2)	3 (100)	32 (88.9)	
Not response	2 (16.7)	0	0	2 (5.6)	

There were 12 patients with clinical symptoms after the first dose of vaccines, of which 2 patients with a history of cancer were treated with an immune checkpoint inhibitor [35]. Case 4 [28] had a history of Sjogren's syndrome and rheumatoid arthritis. Case 6 [30] had a history of on-demand lansoprazole administration. Case 11[35] had a history of taking non-steroid anti-inflammation drugs (NSAIDs) for muscle pain. In addition to leukocyturia and proteinuria, case 8 [32] had a symptom of vascular purpura, with multiple necrotic lesions involving the hands, arms, thighs, and feet and a skin biopsy suggesting leukocytoclastic vasculitis. Follow-up data were obtained for all patients, and 9 of them responded well to treatment. Case 12 [35] was admitted to the hospital with pneumonia-infected shock at month 5 of follow-up and died of multiorgan failure. Case 5 [29] developed AKI and nephrotic proteinuria after the first dose of the Moderna vaccine and went on to receive the second dose of the vaccine. Kidney biopsy was diagnosed as AIN with IgA nephropathy, and he was treated with steroid pulse therapy, but the disease progressed without responding. Case 4 [28] relapsed after one month of steroid discontinuation, but symptoms were quickly controlled with the reintroduction of steroids.

Twenty-one patients developed clinical symptoms after the second dose of the vaccines, with a median onset time of 7 (1–82) days. Of the 21 patients, three had a history of neoplastic disease, including hepatocellular carcinoma, prostate cancer, and diffuse large B-cell lymphoma [34, 36, 42]. Three cases had a history of autoimmune disease, including inflammatory bowel disease, rheumatoid arthritis, and systemic lupus erythematosus [36, 42]. Follow-up data were obtained for all patients, and they responded well to treatments.

In contrast, only 3 patients developed clinical symptoms after the third dose of the vaccines, with a median onset time of 21 (21–30) days. Case 35 [35] had a history of invasive bladder neoplasm and responded well to conservative treatment after discontinuation of Pembrolizumab. The other two patients were treated with immunotherapy and responded well.

## Discussion

### AIN and COVID-19 Vaccination

It was well known that drugs were the most common cause of AIN, especially NSAIDs [47], and currently, the most widely accepted mechanism of drug-induced AIN was the cell-mediated type IV hypersensitivity theory. However, the exact pathophysiological mechanism remained to be elucidated [48].

The manufacturing process of inactivated SARS-CoV-2 vaccine is traditionally used for various vaccines such as influenza vaccine, hepatitis A, and hepatitis B, which suggested some similarities in potential side effects. Of the 35 cases we collected, case 8 presented with leukocytoclastic vasculitis after inactivated COVID-19 vaccination, and leukocytoclastic vasculitis with or without renal symptoms have been described as a side effect of several vaccines, such as influenza virus, hepatitis B virus (HBV), Bacillus Calmette-Guerin (BCG), and human papillomavirus (HPV) [49–52], which strongly supported a causal relationship between vaccines and symptomatology, although these observations were poorly documented and the causal relationship remained to be verified. In the reported patients, almost all started days or weeks after vaccination and showed infiltration of monocytes, neutrophils, and eosinophils on renal biopsy and negative immune fluorescence staining for immune deposits, suggesting that they had a predominantly cell-mediated immune response. 54.3% received the BNT162b2 (Pfizer) vaccine, and a meta-analysis demonstrated that anaphylaxis with the Pfizer-BioNTech vaccine was approximately 10 times higher than that associated with all other vaccines [53]. Components in the SARS-CoV-2 vaccines, such as polyethylene glycol, are also known to be immunogenic and can trigger hypersensitivity-like reactions [54]. Eight patients [25, 27, 31, 32, 35, 37, 38, 45] developed leukocyturia after vaccination, and two patients [27, 28] had peripheral eosinophilia. Therefore, we suspected AIN was an allergic reaction triggered by the vaccines. Case 24 [38] was evaluated 2 months after hospital discharge in the Department of Allergy and Clinical Immunology. The lymphocyte transformation test (LTT) was positive, which supported the involvement of T cells and pointed to a type IV hypersensitivity reaction according to the classification of Gell and Coombs [55, 56].

**Fig. 1:**
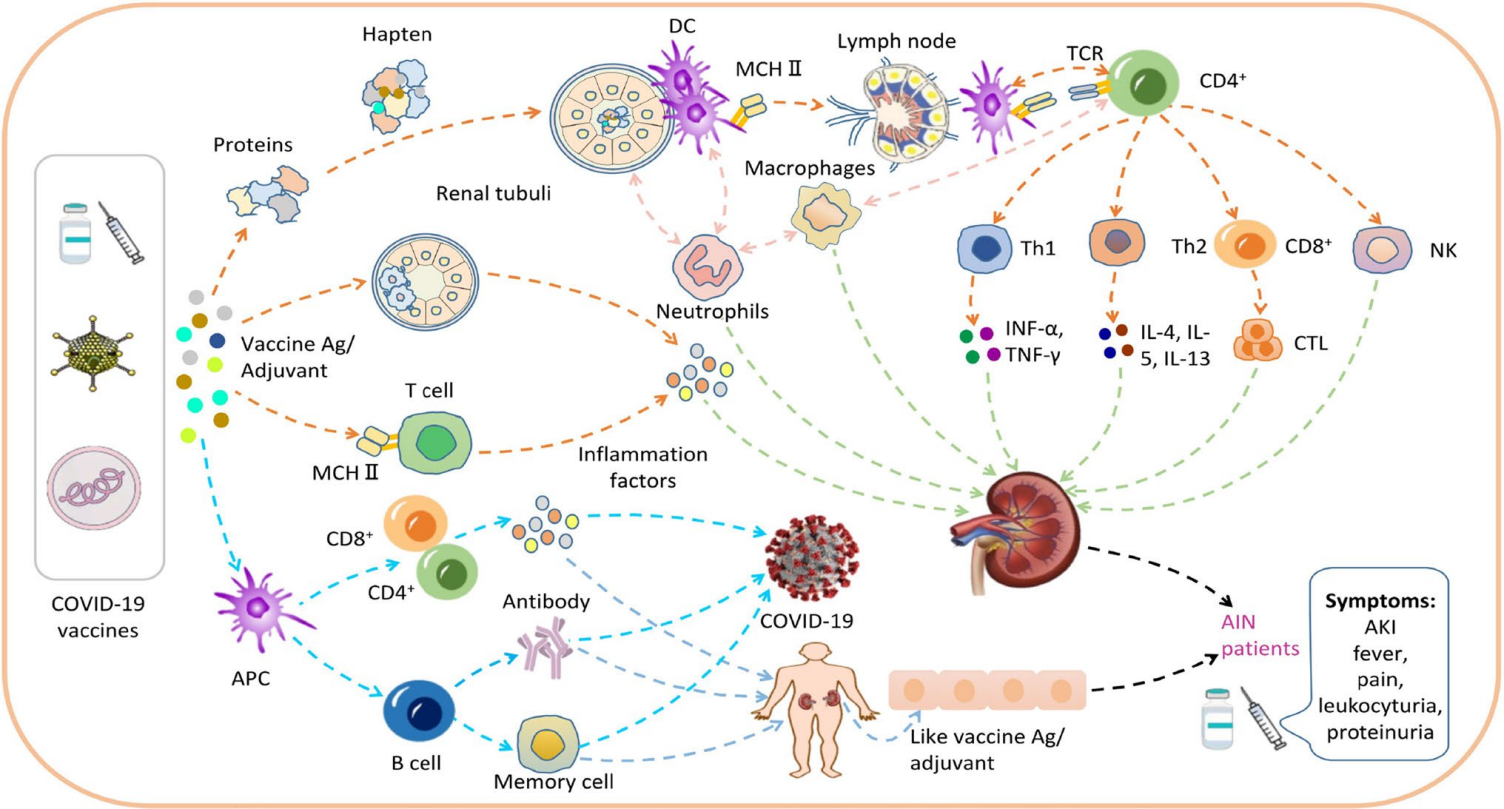
The assumed mechanisms of AIN post COVID-19 vaccination. Hypothesis of AIN caused by COVID-19 vaccination. Haptens: The vaccine may bind to proteins to form protein complexes called "hapten", which are recognized and presented by DCs, causing a subsequent T-cell-mediated toxic response, as well as activation of intrinsic immune cells in the renal stroma, and further amplification of the inflammatory response by crosstalk between different immune cells. P-i concept: some specific structures of the vaccine may stimulate T cells, thus allowing binding to the major histocompatibility peptide complexes and causing inflammatory factor production. Direct injury: vaccines and their products may cause direct renal tubular injury. Molecular mimicry: some structures of vaccines or adjuvants may be homologous to human proteins, and exposure to vaccines triggers antigenic epitopes of cross-reactive antibodies and thus disrupts immune tolerance. The figure refers to the pathogenesis of AIN by Sanchez-Alamo B et al. [48]

The COVID-19 vaccine caused AIN in the form of hapten via a type IV hypersensitivity reaction (Fig. 1). We speculated that vaccines could bind to proteins like drugs to produce immunogenic hapten filtered and endocytosed by peritubular mesangial or tubular epithelial cells [57–60]. These cells acted as antigen presenters, presenting antigenic stimuli to dendritic cells in direct contact with the basal surface of the renal tubular epithelium [61–63]. Once exposed to the antigen or injury signal, normally quiescent dendritic cells were activated and expressed the antigenic compound MHC II molecules in the form of peptides. After that, dendritic cells migrate through the renal lymphatics to regional lymph nodes, where they present antigens to naive T cells, which are activated and migrate to the source of antigenic injury [64–67]. The renal interstitium also contains dormant macrophages and fibroblasts, which are activated and contribute to this initial inflammatory response. This inflammatory response was further amplified by the recruitment of neutrophils, including eosinophils, and was further amplified by bidirectional crosstalk between dendritic cells and T cells or neutrophils [68].

### AIN and SARS-CoV-2 Infection

Infections were known to be a common cause of AIN. Many cases of AIN were caused by infections of the distal kidney, such as hepatitis C virus (HCV) [69], Epstein-Barr virus (EBV) [70], and cytomegalovirus (CMV) [71], which supported a possible causal relationship between SARS-CoV-2 infection and AIN. SARS-CoV-2 has been detected in a kidney allograft associated with a monocyte cell infiltration [17], which suggested that the virus could enter the renal parenchyma and may cause AIN. These findings were supported by recent postmortem histopathological analysis showing positive SARS-CoV nucleoprotein antibody immunostaining in the tubules [72]. In addition, medications were the most common cause of AIN [73, 74]. However, although severely ill COVID-19 patients in intensive care units may be treated with multiple medications, drug-induced AIN was still uncommon in these cases.

Viral infections can disrupt immune tolerance by exposing antigenic epitopes and triggering cross-reactive antibodies [75]. There were numerous reports of antigenic mimicry between viral and human proteins. For example, lupus patients could have immune responses to EBV [76]. Some epitopes of SARS-CoV-2 have been found to exhibit cross-reactivity with autoantigens. Such as respiratory failure [77] and Green-Barre syndrome [78] associated with COVID-19 may be associated with molecular mimicry mechanisms. Similarly, the emergence of Guillain–Barre syndrome (GBS) after influenza vaccination and demyelinating neuropathy after HBV vaccination have been reported [79–81]. Despite the lack of specific evidence for any significant homology between the molecular components of influenza viruses and those in human myelin, molecular mimicry was considered the most likely mechanism. Therefore, we hypothesized that COVID-19 vaccination could induce AIN by the same mechanism.

SARS-CoV-2 infection may also trigger hypersensitivity reactions just as it does after vaccination. Viral and bacterial antigens may trigger cell-mediated injury where they were filtered, concentrated, and secreted in the kidney, accompanied by high blood flow, increasing their exposure and making them targets of immune responses [82, 83]. For example, respiratory viruses can trigger Kawasaki disease [84], while bacterial superantigens of Staphylococcus aureus or Streptococcus pyogenes may lead to toxic shock syndrome [85].

Most patients in the data we collected responded well to steroid therapy, and clinicians should be aware of this renal side effect of SARS-CoV-2 infection. AIN in this condition may be caused not only by the pathogen directly invading the kidney but through different immune mechanisms [86]. Timely diagnosis, including biopsy evaluation, was essential for correct treatment, a good prognosis, and preservation of renal function.

## Conclusion

AIN post SARS-CoV-2 infection and COVID-19 vaccination was rare but with potentially serious complications and satisfactory response to steroid therapy. The pathogenesis, treatment, and long-term prognosis of AIN following SARS-CoV-2 infection and COVID-19 vaccination still need to be further explored.

## Limitations

This review has some limitations. First, the association between AIN and COVID-19 vaccination can only be based on timing and excluding other predisposing factors, such as a history of NSAIDs use in some patients and a history of connective tissue disease (CTD) in others. Second, some reports did not describe a complete etiological study of other causes of AIN. Third, there may be many unreported cases of vaccine-associated AIN, and epidemiological investigations were lacking to determine the true incidence of AIN after vaccination. Fourth, the mechanisms we elucidated need further confirmation. And fifth, due to the small sample size, there may be errors in our statistical analysis.

## Data Availability

Not applicable.
